# Debulking of giant liver hemangiomas with severe symptoms: a case report

**DOI:** 10.1186/s40792-020-00960-4

**Published:** 2020-08-03

**Authors:** Shun-ichi Ariizumi, Yoshihito Kotera, Shingo Yamashita, Akiko Omori, Takaaki Kato, Go Shibuya, Hiroto Egawa, Masakazu Yamamoto

**Affiliations:** Department of Surgery, Institute of Gastroenterology, Tokyo Women’s Medical University, Kawada 8-1, Shinjuku-ku, Tokyo, 162-0054 Japan

**Keywords:** Huge liver hemangiomas, Hepatectomy, 30 cm in diameter, Abdominal fullness

## Abstract

**Background:**

There are no previous reports of debulking of giant liver hemangioma. This report describes our experience with debulking surgery for a patient with bilateral giant liver hemangiomas with severe symptoms.

**Case presentation:**

We present a case of symptomatic giant liver hemangioma in a 41-year-old woman. She presented with abdominal pain and fullness at a local hospital and underwent trans-arterial embolization (TAE). TAE was not effective, and she was not able to walk a long distance. Giant liver hemangiomas, 30 cm and 15 cm in diameter, were located in the right liver and in the left lateral section, respectively, and normal liver parenchyma with tiny liver hemangiomas was present in segment 4. The liver function was normal. However, right hemi-hepatectomy with left lateral sectionectomy was considered to be risky according to the 3DCT volumetry. Therefore, we performed right hemi-hepatectomy in order to reduce her symptoms. The postoperative course was uneventful, and she was discharged on the 14th day after surgery. The abdominal fullness and abdominal pain disappeared immediately after surgery. The hemangiomas in the remnant liver, 15 cm in diameter, showed no change, and she is well without symptoms 7 years after surgery.

**Conclusions:**

Debulking surgery is one of the options for bilateral giant liver hemangiomas with severe symptoms.

## Background

Surgery for liver hemangiomas is still controversial. However, surgical outcomes are reported to be good [1–3]. Giant hemangiomas in the bilateral liver require complicated surgery; therefore, liver transplantation has been suggested as an effective treatment in selected patients [4]. However, the liver function of patients with liver hemangioma is always normal; therefore, we should consider other options for patients with giant liver hemangiomas in the bilateral liver. Several surgeons reported the effectiveness of enucleation of liver hemangioma [1, 2, 4]; however, there has been no report on debulking of liver hemangioma. This report describes our experience with debulking of giant liver hemangiomas in a patient with severe symptoms.

## Case presentation

A 41-year-old woman was referred to our hospital to have treatment for giant liver hemangioma. The liver hemangioma was detected 6 years previously, and the tumor had gradually increased in size starting 3 years before admission. She underwent trans-arterial embolization (TAE) two times for liver hemangioma at another hospital. However, TAE was not effective, and the liver hemangioma increased in size. She felt abdominal pain and abdominal fullness, which made her unable to walk a long distance. There was no past history of chronic hepatitis or blood transfusion. Admission laboratory tests revealed a mildly decreased hemoglobin (10.1 g/dL), normal platelet counts (14.7 × 10^4^/μL), normal serum total bilirubin (1.0 mg/dL), normal serum albumin (4.5 g/dL), normal serum prothrombin time (88%), mildly elevated D-dimer (9.78 μg/mL), and mildly elevated FDP (23.1 μg/mL). The Child-Pugh classification was A, and the indocyanine green retention rate at 15 min was 4%.

**Fig. 1:**
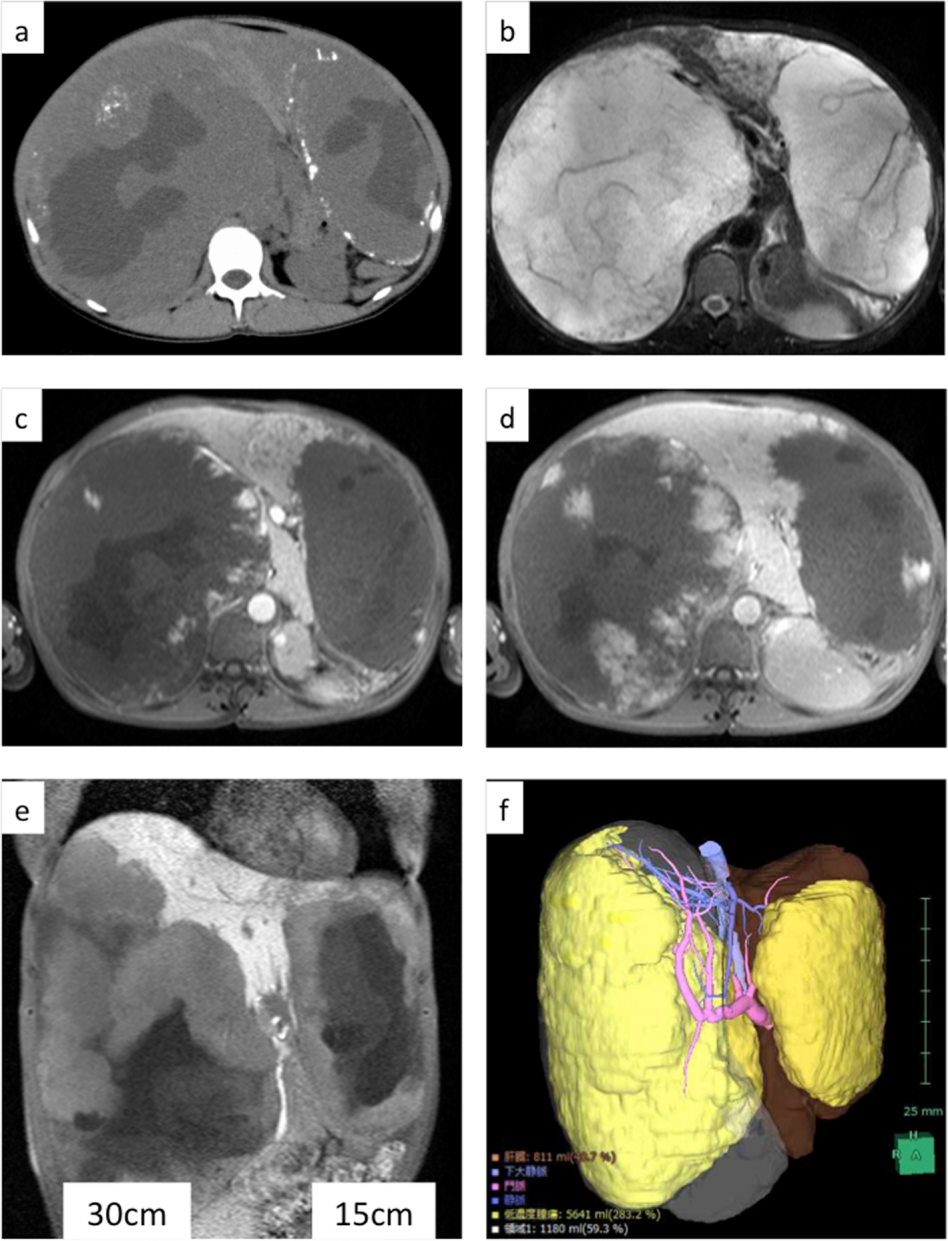
Plain computed tomography (CT) showed giant liver hemangiomas with lipiodol deposits (**a**). Magnetic resonance imaging (MRI) showed a 30-cm liver hemangioma in the right lobe and 15-cm liver hemangioma in the left lateral section (**b** T2 image, **c** arterial phase, **d** portal phase, **e** hepatobiliary image). There is normal liver parenchyma with tiny hemangioma in segment 4. According to 3DCT volumetry, the right liver volume was 60% (**f**)

**Fig. 2:**
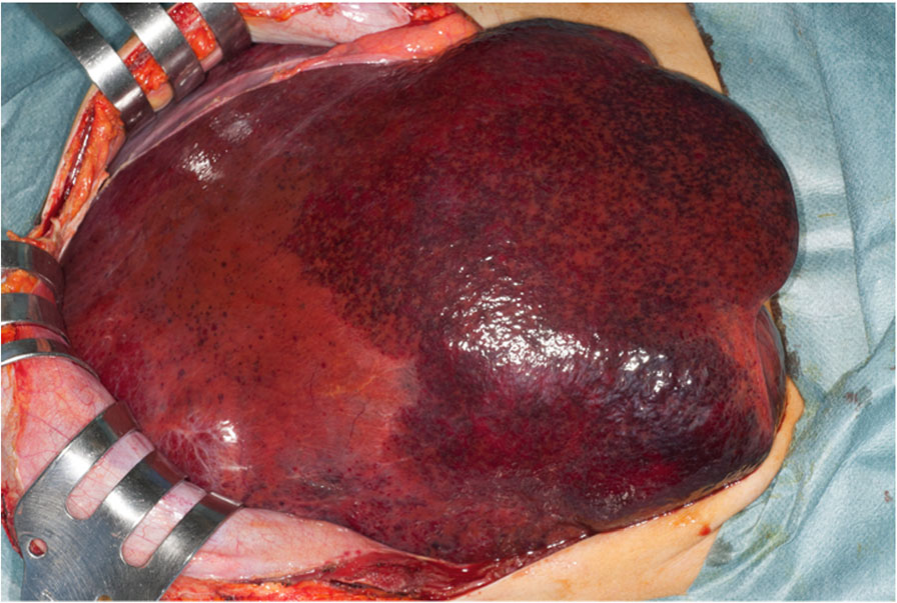
Operative findings show a giant liver hemangioma in the right liver

Plain computed tomography (CT) showed giant liver hemangiomas with lipiodol deposits (Fig. 1a). Magnetic resonance imaging (MRI) showed 30-cm and 15-cm liver hemangiomas in the right liver and left lateral section, respectively (Fig. 1b–e). There were multiple tiny hemangiomas in segment 4. According to 3DCT, right liver volume was 60% and remnant liver volume was 40% (Fig. 1f). We, therefore, concluded that complete resection of both giant hemangiomas was impossible, and we performed debulking right hemi-hepatectomy of giant hemangioma which caused abdominal pain and abdominal fullness (Fig. 2). The total operation time was 4 h, and the total blood loss was 1800 mL. The tumor was a giant hemangioma, 30 cm in diameter, 3.2 kg in weight, and the tumor cells showed cavernous hemangioma on pathological findings. The patient’s postoperative course was uneventful, and she was discharged 14 days after surgery.

**Fig. 3:**
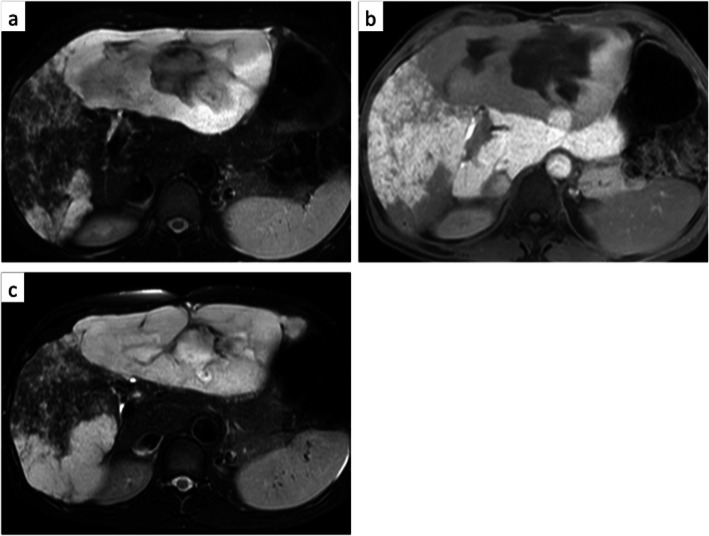
Hemangioma in the remnant liver shows no change, and she is well without symptoms 7 years after surgery (**a** T2 image 3 years after surgery, **b** hepatobiliary image 3 years after surgery, **c** T2 image 7 years after surgery)

The abdominal fullness and abdominal pain disappeared immediately after surgery, and she completed a full marathon 3 years after surgery. The hemangioma in the remnant liver, 15 cm in diameter, shows no change, and she is well without symptoms 7 years after surgery (Fig. 3a–c).

## Discussion

Abdominal pain is the most frequent complaint of liver hemangioma when the tumor is larger than 10 cm in diameter [1–3]. The mechanism of abdominal pain has not been clarified; however, the pressure effect and distention of the liver capsule secondary to infarcts and necrosis might cause pain in cases of giant liver hemangioma. Surgery and non-surgical treatments such as TAE, radio-frequency ablation therapy, and molecular targeting therapy have been reported to be effective to diminish abdominal pain [5]. However, non-surgical treatments are sometimes not effective to relieve symptoms for patients with giant liver hemangioma such as in the case reported here.

Liver function is normal in most cases of liver hemangioma; therefore, liver resection is considered one of the options if it is resectable. However, there are some unresectable cases due to the location of the tumors or coagulopathy. Liver transplantation should be considered at that time [4]. However, because there is a donor shortage problem while the liver function of most patients is normal, other surgical options should be considered. Some patients with polycystic liver disease (PLD) have conditions similar to those of patients with giant liver hemangioma, such as massive hepatomegaly which causes pain and compression of the adjacent organs and affects the patients’ performance status and quality of life. Recently, the treatment strategy for patients with PLD has been reported. Schnelldorfer et al. classified PLD patients with normal liver function according to clinical and radiographic findings into 4 groups, and they recommended hepatic resection with cyst fenestration for PLD if at least a single sector could be preserved [6]. In our present patient, the surgical strategy of PLD was useful because there were no previous reports of debulking of giant liver hemangioma. Therefore, we performed debulking right hemi-hepatectomy because preserving the normal liver parenchyma in segment 4 was thought to be possible based on the MRI findings and 3DCT volumetry.

Most reports of debulking surgery are for advanced ovarian cancer to improve survival after surgery. Very few reports on debulking surgery for benign tumors have been published. Debulking of a colon hemangioma with Kasabach-Meritt syndrome was reported in 1998. In that report, a 39-year-old man had a history of multiple hemangiomas since birth, with involvement of his left perineum and lower extremity, liver, spleen, descending colon, and rectum. He experienced eight episodes of major lower gastrointestinal bleeding since age 18. He underwent left colectomy because a technetium 99m pertechnetate-labeled red cell scan indicated the location of massive bleeding to be from the hemangioma of the sigmoid colon. The patient’s coagulation profile normalized 6 weeks after surgery, and he had not had any further episodes of bleeding 4 years after surgery [7]. A search of the PubMed database was conducted with the keywords “debulking” and “liver hemangioma” between 1975 and 2020. However, the search for reports of debulking of giant liver hemangioma on PubMed yielded no results.

Liver hemangioma is a benign disease; therefore, surgical intervention should be carefully considered when a tumor is increasing in size, larger than 10 cm in diameter, and symptomatic and has coagulopathy. However, liver resection is considered one of the options for a resectable liver hemangioma because short- and long-term surgical outcomes are good. Even if curative resection is not possible, debulking surgery should be considered because surgery improves symptoms and coagulopathy. However, this is a case report and there are no similar reports in the PubMed database. We need more evidence on whether debulking surgery could be acceptable for patients who cannot undergo complete resection of complicated liver hemangiomas.

## Conclusions

Debulking surgery is one of the options for bilateral giant liver hemangiomas with severe symptoms.

## Data Availability

The authors declare that all the data in this article are available within the article.

## Abbreviations

TAE: Trans-arterial embolization
MRI: Magnetic resonance imaging
PLD: Polycystic liver disease
